# A brief body scan mindfulness practice has no positive effect on the recovery of heart rate variability and cognitive tasks in female professional basketball players

**DOI:** 10.3389/fpsyg.2023.1196066

**Published:** 2023-05-31

**Authors:** Dicle Aras, Aysberg Samil Onlu, Tugay Durmus, Caner Cengiz, Damla Guler, Yagmur Guler, Alkan Ugurlu, Monira I. Aldhahi, Mehmet Gülü

**Affiliations:** ^1^Department of Coaching Education, Faculty of Sport Sciences, Ankara University, Ankara, Türkiye; ^2^Ankara University Graduate School of Health Sciences, Movement and Training Sciences Master's Program, Ankara, Türkiye; ^3^Department of Physical Education and Sports, Faculty of Sport Sciences, Ankara University, Ankara, Türkiye; ^4^Department of Recreation, Faculty of Sport Sciences, Yalova University, Yalova, Türkiye; ^5^Department of Physical Education and Sports Education, Faculty of Sport Sciences, Akdeniz University, Antalya, Türkiye; ^6^Department of Rehabilitation Sciences, College of Health and Rehabilitation Sciences, Princess Nourah bint Abdulrahman University, Riyadh, Saudi Arabia; ^7^Department of Sports Management, Faculty of Sport Sciences, Kirikkale University, Kirikkale, Türkiye

**Keywords:** basketball, cognitive task, heart rate variability, Go/No-Go test, mindfulness, NASA TLX, RPE

## Abstract

**Introduction:**

In this study, we examined the acute effects of a short video-based body scan mindfulness practice on the heart rate variability (HRV) and cognitive performance of professional female basketball players after the first half of a simulated basketball game.

**Methods:**

In this crossover randomized controlled trial, nine professional athletes completed a physical loading protocol on two separate days. The protocol consisted of a 10-min Yo-Yo Intermittent Recovery Test Level 1 in the first quarter, followed by a 10-min basketball game in the second quarter. Immediately afterward, they were asked to engage in a 10-min mindfulness practice or watch a 10-min nature-based documentary as a type of mental intervention. Their HRV, Rating of Perceived Exertion (RPE), National Aeronautics and Space Administration Task Load Index 2 (NASA TLX-2), and Go/No-Go test scores were recorded immediately before and after the physical loading and after the mental intervention.

**Results:**

The physical demand, effort, and frustration level subscales of the NASA TLX-2 and the RPE scores were found to be significantly higher after the physical loading, and they returned to the baseline level after both types of mental intervention. The Go/No-Go test scores did not differ depending on the measurement time. All time- and frequency-domain heart rate variability parameters, except the low-to-high frequency ratio, were found to be significantly high immediately after the physical loading protocol. However, these parameters returned to their initial levels after both types of mental intervention.

**Discussion:**

Completing the tests involved in the study protocol successfully induced physical fatigue, as evidenced by consistent measurement tools, but the one-time and short-term mindfulness practice had no additional benefits for the recovery of heart rate variability, cognitive tasks, or subjective assessment methods, such as RPE and NASA TLX-2, in basketball players with no previous experience of mindfulness practice.

## 1. Introduction

Basketball is a sport that involves frequent short-term and short-distance accelerations and decelerations, explosive changes of direction, jumps, and player-to-player contacts, all of which can increase the risk of musculoskeletal injuries during training and competitions (Montgomery et al., 2008). Basketball players compete at an average physiological intensity above the lactate threshold and 85% of their maximum heart rate during a 40-min match (Stojanović et al., 2018). This sport also has one of the longest seasons in professional sports, which can further exacerbate the physical demands and physiological stress placed on athletes during both pre-season and competition periods. These high-intensity movement demands and physiological stressors can result in fatigue (Klusemann et al., 2013). Fatigue is a multidimensional concept that can be defined as a decrease in maximum performance (Coutts et al., 2007; Kellmann, 2010; Knicker et al., 2011; Joyce and Lewindon, 2014). Physical fatigue is characterized by a decrease in strength/power output in muscle cells and motor units, a reduction in muscle performance, and a deterioration in quick decision-making and technical and motor skills (Knicker et al., 2011). However, the effect of fatigue on sport performance is not solely limited to the neuromuscular system (Van Cutsem et al., 2017), and cognitive fatigue is also recognized as a predominant factor (Weinberg and Gould, 2019). Cognitive fatigue is defined as a psycho-biological condition that results from prolonged cognitive load (Desmond and Hancock, 2001; Job and Dalziel, 2001), and it can lead to altered attentional focus, slower reaction times, increased errors in reactions, and decreased use of visual cues in athletes, especially in high-intensity and intermittent sports such as basketball (Baker et al., 2015; Van Cutsem et al., 2017). Since both physiological and cognitive factors play a decisive role in fatigue (Bühlmayer et al., 2017), the speed and efficiency of recovery are important parameters for basketball players (Alaphilippe et al., 2012).

A mindfulness-based regimen is a self-regulatory approach in which a person is fully conscious of their moment-to-moment experiences and actively engages with the present moment without judgment (Kabat-Zinn, 2003; Creswell, 2017). Mindfulness practices involve paying attention to the body and becoming aware of all the various sensations and thoughts that arise during actions (Neale, 2006). Since mindfulness is associated with high levels of wellbeing (Brown and Ryan, 2003), it has been used in various fields, including sports, to enhance team cohesion, reduce anxiety and stress, prevent burnout by reducing fatigue, and mitigate the negative effects of previous adverse situations by promoting present-moment awareness (Kee, 2019; Li et al., 2019). A systematic review study stated that chronic mindfulness practice can enhance sports performance (Sappington and Longshore, 2015). Similarly, in a meta-analytical review, Bühlmayer et al. (2017) reported that mindfulness practice or mindfulness-based interventions have beneficial effects on athletic parameters and can reduce physiological stress in athletes. Macdonald and Minahan (2018) investigated the effects of 8 weeks of mindfulness training on cortisol levels in highly trained wheelchair basketball players and found a decrease in cortisol levels. They suggested that mindfulness training could be helpful in sports. However, all of these studies examined the chronic effects of mindfulness, and the studies examining the acute effects are scarce. A study by Wolch et al. (2021) investigated the effects of brief mindfulness practices on free-throw shooting performance in young basketball players and found no significant difference in performance. In a similar study, Shaabani et al. (2020) observed that brief mindfulness interventions had a beneficial impact on the free-throw performance of basketball players by mitigating the effects of ego depletion (Shaabani et al., 2020). However, due to the limited number of studies and differences in the protocols applied, further studies are needed to better understand the effects of mindfulness on sport performance.

The effects of both physical and mental fatigue could be measured. From a physiological perspective, the optimal level of variability, flexibility, and adaptability observed in the fundamental regulatory systems of the human body is considered an indicator of healthy functioning and overall wellbeing (Shaffer et al., 2014). Heart rate variability (HRV) is frequently used as a non-invasive method that provides information concerning the functioning of the autonomic nervous system (Anon, 1996). Thus, HRV measurements have been used in the field of exercise and sports to calculate heart-based risks, determine stress levels created by training loads, examine the recovery process after loading, and adjust the intensity of the training load (Aras et al., 2022). Studies on the relationship between mindfulness and HRV have also shown that both one-time and multiple-applied mindfulness practices have positive effects on HRV (Ditto et al., 2006; Krygier et al., 2013).

Even though it has been reported in the literature that mindfulness practice has positive chronic effects on athletes (Sappington and Longshore, 2015; Bühlmayer et al., 2017), the number of studies examining its acute effects on the recovery parameters of basketball players is limited. Therefore, the purpose of the present research was to investigate the acute effects of a 10-min body scan mindfulness practice on HRV, cognitive task performance, and subjective scales, as measured by the National Aeronautics and Space Administration Task Load Index 2 (NASA TLX-2) and the Rating of Perceived Exertion (RPE), after the first half of a simulated basketball game in professional female basketball players. According to the general approach in the literature, it was expected that a brief body scan mindfulness practice would result in a greater recovery of HRV parameters and lead to a reduction in both the number of errors and the duration of cognitive work when compared to the control situation.

## 2. Materials and methods

### 2.1. Study design

In this crossover randomized controlled trial, the acute effect of a 10-min mindfulness practice on the recovery of HRV and on cognitive functions was examined. Ethical approval was obtained from the Ankara University Institutional Ethical Committee (2022-SBB-0443). All participants visited the measurement site on three separate occasions. During the first visit, which was a paid familiarization session, the measurement process was explained, and informed consent forms were collected from the participants.

**Figure 1:**
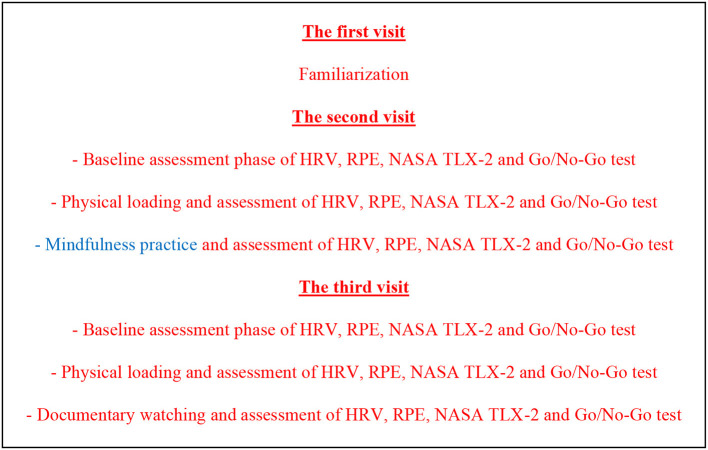
Study design process.

On the second visit, the HRV measurement was conducted. The HRV device remained attached to the person throughout the day during all measurements. Immediately before the physical loading phase, they were given the Rating of Perceived Exertion (RPE) and the National Aeronautics and Space Administration Task Load Index 2 (NASA TLX- 2). Then, they performed a 10-min Yo-Yo Intermittent Recovery Test Level 1 in the first quarter. After 1 min of passive rest, a basketball game was played in the second quarter. When they finished the physical loading phase, they again filled out the RPE and NASA TLX-2 and performed the Go/No-Go test. At the end of the tests, they attended the mindfulness practice or documentary-watching program. In the third meeting, the same process was repeated, only changing the mindfulness practice or documentary-watching phase (Figure 1).

### 2.2. Participants

A total of nine female professional basketball players with a mean age of 20.83 ± 0.75 years, a body weight of 64.62 ± 2.52 kg, body height of 169.00 ± 6.57 cm, a body mass index of 22.70 ± 1.84 kg/m^2^, and a percentage body fat 26.07 ± 2.81 participated in the research voluntarily. The participants were included only if they performed actively in a professional league and regularly attended training sessions 3 days a week for at least the last 3 months. Subjects with any chronic disease such as diabetes mellitus, heart disease, arthritis, hypertension, or musculoskeletal injury in the muscle, ligaments, bones, cartilage, or tendons were excluded from the study. Participants who practiced mindfulness or meditation in their daily lives were also excluded from the study so that the one-time effect could be investigated objectively.

### 2.3. Procedures

#### 2.3.1. HRV measurement

All HRV data were collected using a Polar H10 heart rate monitor (Polar, Finland). The HRV monitors were placed on the chests of the subjects and were not removed until the measurement was finished. The HRV data were recorded using the Elite HRV 5.5.5. application and converted into quantitative HRV data using Kubios HRV Standard 3.5.0. software. The parameters used in the present research were the interval between two R-peaks of the QRS complex in ms (mean RR), mean HR, the standard deviation of NN intervals (SDNN), the root mean square of successive RR interval differences (RMSSD), the baseline width of the RR interval histogram displaying NN intervals (TINN), high frequency (HF), low frequency (LF), and the LF: HF ratio. These parameters were recorded before the physical loading, after the physical loading, and after the mental intervention. All the data were collected between 3 and 5 p.m. The participants were also warned not to exercise or consume alcohol within the 24 h before the test. Participants' caffeine intake was also limited within the previous 12 h of the test.

#### 2.3.2. Go/No-Go test

The Go/No-Go test, each containing 25 stimuli, was administered to all participants three times in a row. The total application time and number of errors were recorded. The tests were carried out on the computer (https://www.psytoolkit.org/experiment-library/experiment_go-no-go.html) after the physical loading and mental intervention. According to the test rules, the participants were asked to follow the warnings on the screen and press the space key as soon as they saw the green (Go) warning. When they saw the red (No-Go) warning, they were asked to wait. Since basketball players must adapt to changing situations in a very short time in all offensive and defensive movements, the Go/No-Go test was used to determine cognitive functions in the current research because of its structural similarity.

#### 2.3.3. RPE and NASA TLX-2

As subjective evaluation methods, RPE and NASA TLX-2 measurements were applied three times: before the physical loading, after the physical loading, and after the mental intervention. The RPE was evaluated using a 6–20 scale (Borg, 1982). The NASA TLX-2 included mental demand, physical demand, temporal demand, performance, effort, and frustration level subscales (Hart and Staveland, 1988). The participants were asked to fill in a scale consisting of 5-point intervals to evaluate their work on these sub-headings, and scores from 0 to 100 were recorded for each subscale.

#### 2.3.4. Physical loading

The physical loading process consisted of two parts. In the first quarter, to cause physical fatigue through intense intermittent exercise, players performed a 10-min Yo-Yo Intermittent Recovery Test Level 1 (Bangsbo et al., 2008). The test started with a speed level of 10 km/h and increased progressively for 10 min. After the completion of the Yo-Yo test, the participants were allowed to rest for 1 min. In the second quarter, the participants played a 10-min basketball game. The aim of the second part of the physical loading process was to induce physical fatigue among the female players and increase their cognitive fatigue by challenging them with quick and demanding decision-making tasks.

#### 2.3.5. Mental intervention

As a mental intervention, a 10-min video-based mindfulness practice was carried out. The practice was recorded by an international yoga, meditation, and mindfulness instructor. Body scan mindfulness practice was based on body awareness and breathing control. The players watched a nature-based documentary for 10 min as a control for mindfulness practice on the other measurement day. In both interventions, videos were projected onto the wall in the locker room of the gym to simulate the match environment. The subjects were asked to sit in the most comfortable position possible. They were also asked to follow all directions and not speak during the interventions.

#### 2.3.6. Mindfulness state

The participants' own assessments were used to determine the state of mindfulness. They were asked to grade the following questions on a five-point Likert scale: (1) I focus on my breathing and (2) I feel in touch with my body (Hafenbrack et al., 2014). The means of the two expressions were compared.

### 2.4. Statistical analyses

All analyses were performed using SPSS v.22 software (SPSS Inc., Chicago, IL, USA). After calculating the descriptive statistics, the distribution and homogeneity of the data were tested. The Shapiro–Wilk test was conducted to evaluate the normality distribution as the number of subjects was below 50. Repeated Measures ANOVA or Friedman tests were used to compare the mean differences at different times, depending on the distribution. Bonferroni for pairwise comparisons was utilized. For in-group statistics, the Friedman test was performed, and the mean difference was calculated using either the paired samples *t*-test or the Wilcoxon test. An alpha value of 0.05 was accepted for all statistical analyses.

## 3. Results

In accordance with the state of mindfulness results, both questions were significantly different after the 10-min mindfulness practice. The participants graded the first question after DW as 1.5 ± 0.55 and after MP as 4.17 ± 0.75 (*p* < 0.026). They answered the second question after DW 1.33 ± 0.52 and after MP 4.33 ± 0.52 (*p* < 0.026).

There was no significant difference between the groups in HRV values, depending on the physical or mental intervention (Table 1). However, all parameters, except the LF: HF value, showed that the sympathetic effect increased significantly after the physical loading. The mean RR in DW was lower after the physical loading than before the physical loading (0.008) and after the mental intervention (0.030). Similarly, the same results were obtained in MP (0.010 and 0.012, respectively). The mean HR was also higher than the first measurement (0.003, 0.028) in DW and in MP (0.028, 0.002). The RMSSD and SDNN measurements taken after the physical loading were lower than the recordings before the physical loading and after the mental intervention in DW (0.028 for RMSSD; 0.018 and 0.024 for SDNN, respectively) and in MP (0.013 and 0.028 for RMSSD; 0.001 and 0.028 for SDNN, respectively). In addition, the measurement observed after the mindfulness practice was significantly lower than the measurement taken before the physical loading in RMSSD and SDNN (0.046 in both). The TINN was observed to be lower after the physical loading compared to the values taken before and after the mental intervention in the DW and MP groups (0.010 and 0.015; 0.012 and 0.004, respectively).

**Table 1:** The HRV test results and their mean comparisons.

		**Before physical loading**	**After physical loading**	**After mental intervention**	***P_***	***n^2^***
Mean RR	DW	689.50 ± 89.41	420.83 ± 54.63^t^	612.50 ± 85.77	0.000^**^	0.994
	MP	706.33 ± 157.34	396.33 ± 42.53^t^	586.17 ± 81.92	0.000^**^	0.982
	*P_*	0.824	0.406	0.598		
Mean HR	DW	88.33 ± 12.01	144.50 ± 18.29^t^	99.83 ± 16.09	0.009^**^	0.995
	MP	90.00 ± 27.44	152.83 ± 16.98^t^	103.83 ± 14.52	0.006^**^	0.982
	*P_*	0.522	0.406	0.631		
SDNN	DW	47.53 ± 17.93	8.08 ± 5.97^t^	39.20 ± 14.42	0.000^**^	0.928
	MP	54.60 ± 24.4	8.15 ± 4.68^t^	35.32 ± 13.54	0.006^**^	0.882
	*P_*	0.337	0.983	0.641		
RMSSD	DW	36.18 ± 20.8	5.63 ± 5.24^t^	22.50 ± 8.87	0.006^**^	0.876
	MP	40.92 ± 24.22	3.60 ± 0.51^t^	15.30 ± 5.52	0.030^*^	0.844
	*P_*	0.724	0.748	0.109		
TINN	DW	197.67 ± 63.3	35.00 ± 30.9^t^	193.00 ± 65.45	0.000^**^	0.953
	MP	249.50 ± 112.71	29.57 ± 15.25^t^	166 ± 65.06	0.000^**^	0.883
	*P_*	0.349	0.707	0.490		
HF	DW	465.00 ± 335.3	4.67 ± 4.84^t^	204.83 ± 120.12	0.019^*^	0.733
	MP	667.00 ± 485.33	2.17 ± 1.72^t^	111.83 ± 93.61	0.020^*^	0.704
	*P_*	0.421	0.261	0.166		
LF	DW	1,360.50 ± 858.59	39.50 ± 43.2^t^	1,310.50 ± 1,413.89	0.006^**^	0.642
	MP	2,408.83 ± 1,583.29	41.83 ± 45.78^t^	947.83 ± 527.31	0.006^**^	0.780
	*P_*	0.184	0.929	0.873		
LF: HF	DW	3.92 ± 0.95	12.31 ± 13.35	6.34 ± 2.88	0.234	
	MP	6.71 ± 3.30	15.03 ± 15.38	7.12 ± 2.55	0.309	
	*P_*	0.074	0.750	0.630		

DW, documentary watching; MP, mindfulness practice; SDNN, standard deviation of NN intervals. RMSSD, root mean square of successive RR interval differences; TINN, baseline width of the RR interval histogram displaying NN intervals; HF, high frequency; LF, low frequency; LF:HF, LF: HF ratio. ^*^p < 0.05, ^**^p < 0.01.

***P_***: The ***P_*** value in the column represents the average comparison of the values obtained at different times in a single group. The ***P_*** value in the row represents the average comparison of two different groups.

^t^indicates the measurement time at which the difference originates.

Frequency-domain parameters also showed close results. The HF was recorded lower after the physical loading than before the physical loading and after the mental intervention in DW (0.020, 0.009, respectively) and MP (0.020, 0.034, respectively). Additionally, there was also a significant change between the first and last HF values in both mental intervention groups (0.043 in the DW group and 0.024 in the MP group). Finally, the LF values followed a similar pattern, with exposure to physical loading resulting in lower LF values in both intervention groups compared to before physical activity levels and after mental intervention levels (0.014 and 0.028 for the DW group; 0.042 and 0.021 for the MP group).

When the Go/No-go test results were examined, no significant difference was found between the groups or in-group results depending on physical loading (Table 2).

**Table 2:** The Go/No-go test results and their mean comparisons.

		**Before physical loading**	**After physical loading**	**After mental intervention**	***P_***
Test duration	DW	45.71 ± 3.11	46.67 ± 2.66	47.20 ± 3.68	0.731
	MP	44.85 ± 2.14	46.11 ± 3.89	44.55 ± 4.17	0.364
	*P_*	0.318	0.764	0.268	
Number of errors	DW	1.94 ± 1.90	2.17 ± 1.17	1.83 ± 1.17	0.739
	MP	2.02 ± 2.63	2.00 ± 1.67	3.17 ± 2.14	0.272
	*P_*	0.417	0.845	0.240	

DW, documentary watching; MP, mindfulness practice.

***P_***: The ***P_*** value in the column represents the average comparison of the values obtained at different times in a single group. The ***P_*** value in the row represents the average comparison of two different groups.

Table 3 shows representation of RPE and NASA TLX-2 scores. No RPE scores differed significantly in the subjective measurements used in the study. However, there were differences in RPE and some NASA TLX-2 scores, depending on the application time. The RPE score recorded after the physical loading was higher than that before the physical loading and after the mental intervention in both the DW (0.000 and 0.001, respectively) and MP groups (0.003 and 0.003, respectively). The NASA TLX-2, physical demand score, was higher after the physical loading than before the physical loading and after the mental intervention in the DW (0.002) and MP groups (0.027 and 0.026, respectively). The effort score was again higher after physical loading than pre-physical loading and post-mental intervention in the DW (0.002) and MP groups (0.006 and 0.028, respectively). Finally, in the DW group, frustration levels were recorded to be higher after the physical loading than before the physical loading (0.004) and after mental intervention (0.035). This parameter was also found to be significantly higher after physical loading (0.025) in the MP group.

**Table 3:** The results of the RPE and NASA TLX-2 subscales and their mean comparisons.

		**Before physical loading**	**After physical loading**	**After mental intervention**	***P_***	***n^2^***
RPE	DW	6.00 ± 0.00	13.67 ± 2.16^t^	7.00 ± 0.89	0.000^**^	0.995
	MP	6.00 ± 0.63	14.17 ± 3.60^t^	6.00 ± 0.42	0.003^**^	0.985
	*P_*	1.000	0.777	0.051		
Mental demand	DW	19.17 ± 12.01	35.83 ± 24.58	31.67 ± 19.41	0.121	
	MP	15.83 ± 12.81	40.83 ± 30.07	35.83 ± 35.41	0.385	
	*P_*	0.490	0.759	0.806		
Physical demand	DW	5.83 ± 2.04	66.67 ± 20.90^t^	5.00 ± 0.00	0.000^**^	0.912
	MP	6.67 ± 4.08	70.00 ± 30.17^t^	6.67 ± 2.59	0.005^**^	0.883
	*P_*	0.902	0.462	0.138		
Temporal demand	DW	25.83 ± 35.27	54.17 ± 27.10	36.67 ± 35.02	0.118	
	MP	30.00 ± 38.73	55.00 ± 28.64	30.83 ± 29.23	0.327	
	*P_*	0.849	0.686	0.871		
Performance	DW	48.33 ± 47.50	60.83 ± 15.63	43.33 ± 43.55	0.161	
	MP	35.83 ± 34.70	61.67 ± 27.51	34.17 ± 32.93	0.154	
	*P_*	0.806	0.746	0.690		
Effort	DW	9.17 ± 4.92	75.83 ± 17.15^t^	12.50 ± 11.73	0.000^**^	0.896
	MP	12.50 ± 8.22	70.00 ± 33.47^t^	16.67 ± 19.15	0.007^**^	0.849
	*P_*	0.503	0.712	0.858		
Frustration level	DW	13.33 ± 10.33	53.33 ± 16.02^t^	28.33 ± 38.43	0.005^**^	0.787
	MP	25.00 ± 32.25	51.67 ± 29.27^t^	15.83 ± 12.01	0.048^*^	0.714
	*P_*	0.566	0.905	0.464		

RPE, the rating of perceived exertion. ^*^p < 0.05, ^**^p < 0.01.

***P_***: The ***P_*** value in the column represents the average comparison of the values obtained at different times in a single group. The ***P_*** value in the row represents the average comparison of two different groups.

^t^indicates the measurement time at which the difference originates.

## 4. Discussion

This study was intended to investigate the acute effects of a brief mindfulness practice on HRV, cognitive task performance, and self-perceived physical exertion in professional female basketball players. For this purpose, the participants underwent a 10-min video-based body scan mindfulness intervention immediately after half-time during a simulated basketball game. To fulfill the purpose, the authors administered two different mental interventions: mindfulness practice (MP) and documentary watching (DW).

As a result, it was observed that RPE increased after physical fatigue and reached its baseline level after the passive recovery phase, regardless of the type of MI. In this case, mindfulness practice had no additional effect on RPE. Similar results were also observed on some subscales of the NASA TLX-2 inventory. The subscales for mental demand, temporal demand, and performance remained unchanged, whereas the subscales for physical demand, effort, and frustration increased following physical fatigue and subsequently returned to their baseline levels after MI, regardless of the type of MI. In this study, DW or MP had almost the same effects on an increased rating of perceived exertion, perception of physical demand, effort, and frustration level among the participants. Another factor that was measured in the study was the state of mindfulness, which was used to assess the effectiveness of the mindfulness practice. The results indicate that the intervention was successful in enhancing the participants' mindfulness awareness.

The HRV parameters observed in the current research show that 20 min of physical loading caused physical fatigue. All time- and frequency-domain HRV parameters, except the LF: HF ratio, indicated a clear increase in sympathetic activity. There was a significant decrease in mean RR (0.000 in both), SDNN (0.000 in DW and 0.006 in MP), RMSSD (0.006 in DW and 0.030 in MP), TINN (0.000 in both), HF (0.019 in DW and 0.020 in MP), and LF (0.006 in both) and an increase in mean HR (0.009 in the DW group and 0.006 in the MP group). Moreover, in the LF: HF ratio, an increase was ~215.05% in the DW group and ~123.99% in the MP group, even though the LF: HF ratio was not significant. This proves the consistency of the HRV measurement and the efficiency of the physical loading phase. These results are consistent with those from NASA TLX-2 and RPE. Another remarkable result was that the 10-min passive resting time was adequate to recover almost all of the HRV parameters. The mean RR, mean HR, TINN, and LF reached their initial levels at the end of the 10 min. On the contrary, the SDNN, RMSSD (0.046 in both groups), and HF (0.043 in the DW group and 0.024 in the MP group) values were still lower after MI compared to the first measurement taken before PL in both groups. This result indicates that a longer time may be needed for some HRV parameters to recover. Besides, the most important finding was that the results were not explicitly affected by the type of MI. No significant difference was found in any of the HRV values obtained from the participants after MP and DW.

Previous research has suggested that mindfulness practices are effective for the autonomic nervous system and should be examined in more detail (Krygier et al., 2013). Since HRV has been accepted as an indicator of the autonomic nervous system that is affected by both physical and mental health (Brown et al., 2021), the present research focused on the changes in HRV measurement after PL and MI and its effects on cognitive tasks. As a kind of mindfulness practice, a 10-day Vipassana meditation was carried out by people with no previous meditation history, and its chronic and acute effects on HRV and some wellbeing and ill-being inventories were investigated (Krygier et al., 2013). According to the results, a 10-day mindfulness intervention, which involved 5 min of rest and 5 min of meditation practice, caused an increase in all the wellbeing parameters and decreased many of the ill-being parameters despite no change in HRV. However, the comparison of the resting baseline and mindfulness phases of all sessions revealed a task effect on HF power, indicating the parasympathetic dominance of HRV. The task effect observed may be due to the fact that the MP in this study was based on breathing exercises, in particular, or the repetition of the sessions. Kirk and Axelsen (2020) conducted a study comparing the acute and chronic effects of mindfulness and music-listening interventions on HRV. The daily time commitment for both interventions was 20 min for the first 5 days and 30 min for the last 5 days. At the end of the 10 days, they found that the mindfulness intervention was more effective in improving HRV, including sleep quality. In terms of the acute comparisons, they stated that both practicing mindfulness and listening to music had similar positive effects on HRV compared to the control group. The results obtained from this study can be considered congruent with those in the current study, which revealed that watching a documentary was as effective as mindfulness in improving HRV. Based on this result, we can conclude that any relaxing activity could have acutely positive effects on HRV. However, this similarity shows that, when mindfulness is practiced for a long time, its acute effects can be more pronounced in addition to its chronic effects. Another study supporting this statement was conducted by Delgado-Pastor et al. (2013). They compared 30-min mindfulness with 30-min random thinking. As a result, they found that experienced Vipassana meditators had better HRV values after MP. Delgado-Pastor et al. (2013) also showed that practicing mindfulness regularly has more pronounced effects. In a study in which mindfulness body scan meditation was used as in the current research, it was stated that respiratory sinus arrhythmia, which is accepted as an indicator of vagal activity, increased significantly after 20 min of intervention in people with no meditation history when compared to progressive muscle relaxation or the control group (Ditto et al., 2006). This increase observed in the mindfulness body scan meditation group was similarly high when measured 1 month later. In the measurement repeated 1 month later, the results of the progressive muscular relaxation group were also high. However, in this first part of the research, they found no significant change in systolic and diastolic blood pressure. In the second part of their study, in which the data were collected from different participants, it was found that the LF and HF power of HRV were higher after 20 min of mindfulness body scan meditation in comparison to 20 min of listening to an audiobook, with no change found in the blood pressure. As a result, they concluded that some of the changes were independent of the type of relaxation activity and that this research revealed similarities and differences between mindfulness body scan meditation and other relaxation activities. However, there are also other studies indicating that there is no interaction between HRV and mindfulness. Brown et al. (2021), in the first comprehensive meta-analysis study in which they analyzed the results of 19 randomized controlled trials, claimed that mindfulness-based interventions were not significantly associated with vagally mediated HRV due to the heterogeneity of the studies.

Since many studies have examined the effects of different mindfulness interventions on HRV, it may be useful to mention the comparisons between HRV biofeedback practice and MP in the literature. The closest study to the current research was conducted by Paul and Garg (2012). They discovered positive chronic effects of 10 sessions of 20-min HRV biofeedback training on HRV values such as total HRV, LF, HF, and respiration rate, as well as state-trait anxiety and self-efficacy in basketball players that were measured against 10-min motivational video-watching and the control condition. It was demonstrated that HRV biofeedback training performed by breathing at one's own resonant frequency increases parasympathetic activity by increasing the respiratory sinus arrhythmia and that it is an effective way to enhance the activity of the autonomic nervous system. However, different results were found in different studies examining the effects of HRV biofeedback training on stress and, thus, on the autonomic nervous system when compared to MP. In a long-term study comparing the effects of 6-week mindfulness practice, which was based on breathing, thoughts, feelings, and physiological sensations, and HRV biofeedback on stress levels, no significant difference was found in the experimental groups or in the control group (Brinkmann et al., 2020). In another comparison, Van Der Zwan et al. (2015) investigated the effects of a 5-week physical activity, mindfulness meditation, and HRV biofeedback interventions on reducing stress, depression, and anxiety. They concluded that all interventions were significantly effective, with no differences between the interventions. The duration of both interventions was 10 min/day for the first week, 15 min/day for the second week, and 20 min/day for the remaining weeks, and the MP was focused on breathing, body scanning, and mindful walking. There is another study in which the stress level of basketball players was measured. The authors measured salivary cortisol concentration and immunoglobulin-A secretion as a useful method to monitor physical and psychological stress and reach efficient recovery approaches in well-trained basketball players (Macdonald and Minahan, 2018). They indicated that 8 weeks of mindfulness practice was a beneficial and relaxing method, as it resulted in decreased salivary cortisol concentration during the competition period.

Another purpose of the present study was to investigate the potential positive effects of mindfulness practice (MP) on cognitive performance and to determine how cognitive tasks are affected by MP. For this reason, the subjects were asked to perform the Go/No-go test before and after the PL and after the MI, and the duration of the test and the number of errors were recorded. However, no significant difference was found in cognitive task performance of the participants before and after physical loading, and a lack of change in the mental demand subscale of the NASA TLX-2 confirmed this finding. The lack of improvement in the duration of the Go/No-go test and the number of errors after the mental intervention showed that both MP and DW did not have a significant effect on cognitive performance. This finding should be examined from two different perspectives. First, physical fatigue during the first half of a simulated basketball game may not affect cognitive performance. Second, any mental intervention does not improve cognitive task scores. The practice of mindfulness, which was used as a mental relaxation method, was no different from watching documentaries in terms of improving cognitive task performance. In the literature, there are studies examining the effects of brief mindfulness practices on cognitive tasks and mental fatigue. In a study conducted by Zhu et al. (2020a), the effects of acute mindfulness practice on cognitive functions were examined. The study involved having participants perform a 6-min mindfulness practice during the half-time break of a simulated soccer game using the Loughborough Intermittent Shuttle Test to reflect athletic performance as in a soccer game. The results indicated that, while the control group did not show any change in the Stroop Color and Word Test, the Corsi block-tapping test, or the rapid visual information processing task test, both the CHO and CHO + mindfulness groups exhibited improvements in the Corsi block-tapping test and the rapid visual information processing task test. Only the CHO + mindfulness group showed an increase in the Stroop Color test score. In a similar study, researchers evaluated the same 6-min mindfulness practice on the recovery process during a half-time break of a simulated soccer game (Zhu et al., 2020b). The authors concluded that a 6-min brief mindfulness practice applied with CHO consumption increases mindfulness levels and decreases mental fatigue. They also claimed that CHO + mindfulness intervention increased the repeated sprint ability of the athletes compared to the control or only CHO consumption group. Although these studies are structurally similar to the current one, the results obtained were different. Even though MP was applied for 10 min and the score of the state of mindfulness was significantly higher in the present study, it was not effective in improving cognitive function or reducing physical fatigue. The difference between the studies may have occurred due to CHO consumption. Another study compared the acute effects of hatha yoga practice and mindfulness intervention on cognitive functions and mood (Luu and Hall, 2017). The researchers found that both interventions were effective in improving either cognitive task performance or mood levels at the same levelwhen compared to the control group. Both interventions lasted 25 min in the study, and cognitive task performance was evaluated using the Stroop test and mood using the Profile of Mood States-2 Adult Short. There may be several reasons why this research and the current study show different results. First, the participants were selected from people who regularly practice yoga and meditation. Second, the application time was 25 min. Normally, the effect can be expected to increase as the duration increases. Another reason is that, before the mindfulness intervention, the participants did not engage in tasks that required physical and mental efforts, as is the case in this study. The acute effects of brief mindfulness practice were evaluated on free-throw shooting performance during low- and high-pressure phases in young basketball players (Wolch et al., 2021). The researchers stimulated high-pressure conditions by informing the participants that their shots would be recorded. The comparison of a 15-min audio mindfulness intervention with 15-min audio basketball history listening demonstrated no change in free-throw shooting performance. The only significant effect of a brief MP was observed on cognitive-state anxiety. Their somatic anxiety and self-confidence scores did not differ. In a similar study, Shaabani et al. (2020) evaluated the effects of a 15-min audio mindfulness training on free-throw shooting performance in experienced basketball players. They indicated that free-throw performance, which was impaired by ego depletion by applying the Stroop test for 15 min, could be restored to pre-ego depletion levels through MP. However, another study found no positive effects of a 4-min audio-based mindfulness practice on plank exercise in comparison to 4 min of audiobook listening (Stocker et al., 2019). The participants with no previous mindfulness experience did not show any improvement in cognitive task performance, depending on the MP, after the ego depletion process. It is evident from the results of these studies that the effects of mindfulness practice on sports performance are not certain and need further investigation.

## 5. Conclusion

In this research, the effects of a brief body scan mindfulness practice on HRV, cognitive task performance, and subjective scales in professional female basketball players were evaluated. The study found consistent results across all physiological, cognitive, and subjective measurement tools, suggesting that the results were consistent. Although the participants returned to their baseline levels after the 10-min MI phase, this was not specifically due to MP. According to the results, it could be concluded that acute MP had no additional positive effect on HRV or on cognitive tasks in athletes with no previous mindfulness experience. According to this result, athletes may not benefit acutely from short-term mindfulness practice. Nevertheless, it has been stated that mindfulness intervention has many positive effects on athletes (Sappington and Longshore, 2015; Bühlmayer et al., 2017; Creswell, 2017). In fact, it has been reported that mindfulness can even be effective in reducing sports injuries by reducing the level of anxiety and increasing awareness and attention in young football players (Naderi et al., 2020). However, these positive effects are more pronounced when mindfulness is applied for a long time. For this reason, it is recommended that the practice of mindfulness be continuously included in the training programs of athletes. Particularly in such high-intensity, intermittent sports branches, athletes should make mindfulness practice a habit. While the acute effect may not be as pronounced in athletes with no previous mindfulness experience, maintaining a long-term practice may lead to more significant benefits. Thus, even in basketball, with a recovery interval as short as 10 min, mindfulness can be utilized. Therefore, in future research, the acute effect can be examined in basketball players who consistently practice mindfulness.

Additionally, the results of studies on the acute effects of mindfulness in the literature are not consistent. Having many different mindfulness interventions, different mindfulness practice durations, different control group practices, different ways of practicing mindfulness (video-based, audio-based, and accompanied by an expert), HRV recording time, and different body positions of the participants during the recording of HRV increase the heterogeneity of mindfulness studies. Therefore, more controlled studies are needed. For example, because the effect of respiratory control on the autonomic nervous system is evident, and this is reflected in the measurement of HRV, respiratory rate, and depth, these can also be controlled during mental practices. Apart from this, comparing the acute and chronic effects of different mindfulness practices in future studies may contribute to the development of a standard method. As another suggestion, future researchers may conduct performance tests and more valid physiological tests that also allow the observation of neural changes to monitor the effects of acute mindfulness practice or fatigue, in addition to the cognitive and perceptual measurement methods used in this research. Thus, more precise results can be obtained. The most important limitation of this study was the small number of participants. Subsequent research may increase the number of participants and attempt this protocol not only on women but also on men. Similarly, research can be applied to different age groups, such as children and young athletes.

## Data availability statement

The raw data supporting the conclusions of this article will be made available by the authors, without undue reservation.

## Ethics statement

The Ethical approval was obtained from the Ankara University Institutional Ethical Committee (2022-SBB-0443). The patients/participants provided their written informed consent to participate in this study.

## Author contributions

DA: conceptualization, methodology, software, validation, formal analysis, investigation, resources, supervision, and project administration. AS, TD, CC, DG, YG, and AU: data curation. DA and AU: writing—original draft preparation and visualization. AU, MG, and MA: writing—review and editing. MA: funding acquisition. All authors contributed to the article and approved the submitted version.
